# Gonadal Hormones E2 and P Mitigate Cerebral Ischemia-Induced Upregulation of the AIM2 and NLRC4 Inflammasomes in Rats

**DOI:** 10.3390/ijms21134795

**Published:** 2020-07-07

**Authors:** Pardes Habib, Julie Harms, Adib Zendedel, Cordian Beyer, Alexander Slowik

**Affiliations:** 1 Department of Neurology, Medical Faculty, RWTH Aachen University, 52074 Aachen, Germany; phabib@ukaachen.de; 2 Institute of Biochemistry and Molecular Immunology, Medical Faculty, RWTH Aachen University, 52074 Aachen, Germany; 3 Institute of Neuroanatomy, Medical Faculty, RWTH Aachen University, 52074 Aachen, Germany; julie.harms@rwth-aachen.de (J.H.); azendedel@ukaachen.de (A.Z.); cbeyer@ukaachen.de (C.B.); 4 JARA Brain, RWTH Aachen University, 52074 Aachen, Germany

**Keywords:** Stroke, Inflammasomes, AIM, NLRC4, Estrogen, Progesterone, Neuroprotection, Microglia, Astrocytes, OGD

## Abstract

Acute ischemic stroke (AIS) is a devastating neurological condition with a lack of neuroprotective therapeutic options, despite the reperfusion modalities thrombolysis and thrombectomy. Post-ischemic brain damage is aggravated by an excessive inflammatory cascade involving the activation and regulation of the pro-inflammatory cytokines IL-1β and IL-18 by inflammasomes. However, the role of AIM2 and NLRC4 inflammasomes and the influence of the neuroprotective steroids 17β-estradiol (E2) and progesterone (P) on their regulation after ischemic stroke have not yet been conclusively elucidated. To address the latter, we subjected a total of 65 rats to 1 h of transient Middle Cerebral Artery occlusion (tMCAO) followed by a reperfusion period of 72 h. Moreover, we evaluated the expression and regulation of AIM2 and NLRC4 in glial single-cell cultures (astroglia and microglia) after oxygen–glucose deprivation (OGD). The administration of E2 and P decreased both infarct sizes and neurological impairments after cerebral ischemia in rats. We detected a time-dependent elevation of gene and protein levels (Western Blot/immunohistochemistry) of the AIM2 and NLRC4 inflammasomes in the post-ischemic brains. E2 or P selectively mitigated the stroke-induced increase of AIM2 and NLRC4. While both inflammasomes seemed to be exclusively abundant in neurons under physiological and ischemic conditions in vivo, single-cell cultures of cortical astrocytes and microglia equally expressed both inflammasomes. In line with the in vivo data, E and P selectively reduced AIM2 and NLRC4 in primary cortical astrocytes and microglial cells after OGD. In conclusion, the post-ischemic elevation of AIM2 and NLRC4 and their down-regulation by E2 and P may shed more light on the anti-inflammatory effects of both gonadal hormones after stroke.

## 1. Introduction

Acute ischemic stroke (AIS) is a prevalent disease and has remained a leading cause of disability and mortality worldwide for decades. Although recent advances in stroke reperfusion therapies have remarkably improved clinical outcomes, the absence of effective neuroprotective strategies are associated with high post-stroke mortality rates and severe permanent disabilities [1]. Pathophysiologically, post-ischemic neuroinflammation appears to be a major contributor not only to the temporal progression of the infarction but also to the extent of the final brain damage [2].

In the scope of neuroinflammation, multiprotein complexes termed inflammasomes are attributed to play a crucial role in the initiation and maintenance of the inflammatory cascade, especially in the maturation and secretion of IL-1β and IL-18 [3]. In general, inflammasomes belong to intracellular pattern recognition receptors sensing pathogen-associated molecular patterns (PAMPs) or sterile inflammatory damage-associated molecular patterns (DAMPs) [4]. Upon activation, inflammasomes form multicomplex platforms consisting of the inflammasome receptor, in some cases of the adaptor protein ASC (apoptosis-associated speck-like protein containing a CARD), and a caspase-1, which in turn convert the pro-form of IL-1β or IL-18 into their mature forms [4]. The most investigated inflammasome is NLRP3 (NACHT, LRR, and PYD domains containing protein 3 or cryopyrin), which was also reported to be a key player in maintaining neuroinflammation after cerebral ischemia in rodents. Various studies using different approaches such as gene deletion [5], fasting [6] or antibody application [7] against NLRP3 highlighted a marked improvement of the post-ischemic inflammatory status and motor functions, which significantly reduced the infarct sizes in rodent ischemia models. Regarding the regulation of the NLRP3 inflammasome after cerebral ischemia there are different reports depending on the species. In contrast to the studies using a murine stroke model, we found a reduction of NLRP3 in the brain of rats [8].

Beside NLRP3, other inflammasomes are more frequently the focus of stroke research. The deletion of AIM2 (absent in melanoma 2) and NLRC4 (NLR family CARD domain-containing protein 4) as two candidates proved to be protective after ischemic stroke. Here, the authors subjected NLRC4^−/−^ and AIM2^−/−^ knockout mice to tMCAO and revealed that these both inflammasomes seemed to have a more significant impact on infarct sizes and behavioral and clinical outcomes than NLRP3 [9]. The depletion of NLRP3 failed to display any beneficial effects after stroke in this study. Most recent studies further underlined the role of AIM2 and NLRC4 in the post-ischemic pathophysiology. For instance, treatment with the histone deacetylases 3 (HDAC3) inhibitor reduced infarct sizes, most probably mediated by a downregulation of AIM2 after acute ischemia [10]. Nevertheless, the role of AIM2 or NLRC4 in the context of acute ischemia has been little researched so far. In the aforementioned study of our group, we could show that the mRNA levels of AIM2 and NLRC4 were upregulated after stroke. Still, we did not make any statements about the temporal and cellular distribution of both inflammasomes. Furthermore, it was not known whether the two inflammasomes are regulated by the neuroprotective gonadal steroid hormones 17β-Estradiol (E2) and Progesterone (P) in a similar way to NLRP3.

Numerous studies have successfully demonstrated that E2 and P exert robust neuroprotective effects in in vivo and in vitro cerebral ischemia models [8,11,12,13,14,15]. Both steroid hormones are capable of reducing infarct volumes and improving neurological/clinical outcomes after experimental cerebral ischemia. Furthermore, primary astrocytes or microglia, as well as microglial cell lines, display a higher ischemic/hypoxic tolerance after E2 or P administration. Besides the pleiotropic effects of E2, it is assumed that its cytoprotective actions are mediated by dampening the inflammatory cascade. Several studies in recent years have expanded our understanding of E2-dependent modulation and regulation of neuroinflammation. For example, we know that E2 regulates microglial activation to the extent that it drives these immuno-competent cells to a more “anti-inflammatory” phenotype after ischemia [16]. E2 also appears to have a regulating influence on inflammasomes. For instance, E2 has been shown to significantly reduce NLRP3 inflammasomes in allergic airway inflammation [17] and mediated a marked reduction of inflammation and cancer progression in patients via estrogen receptor beta [18]. Along with E2, progesterone is also attributed a strong neuroprotective role as it influences many detrimental cellular mechanisms such as neuroinflammation by reducing cytokine expression and regulating glial functions [11,12,13,14,19]. However, as with E2, little is known about its impact on inflammasomes and especially on the AIM2 and NLRC4 inflammasomes after ischemic stroke.

Thus, in this study, we intended to investigate the regulation of AIM2 and NLRC4 after cerebral ischemia in rats and to evaluate the impact of the neuroprotective steroids E2 and P on the post-ischemic regulation of the aforementioned inflammasomes. Moreover, we assessed the expression and regulation of AIM2 and NLRC4 in glial single-cell cultures (astroglia and microglia) after oxygen–glucose deprivation (OGD).

We provide evidence that AIM2 and NLRC4 inflammasomes are upregulated in the post-ischemic brains of rats. This increase was significantly attenuated by the gonadal steroid hormones E2 and P after both in vivo and in vitro ischemia, which may contribute to further mechanistic clarification of the anti-inflammatory effect of both steroids.

## 2. Results

### 2.1. E2 and P Decrease Both Infarct Sizes and Neurological Impairments after Cerebral Ischemia in Rats

**Figure 1:**
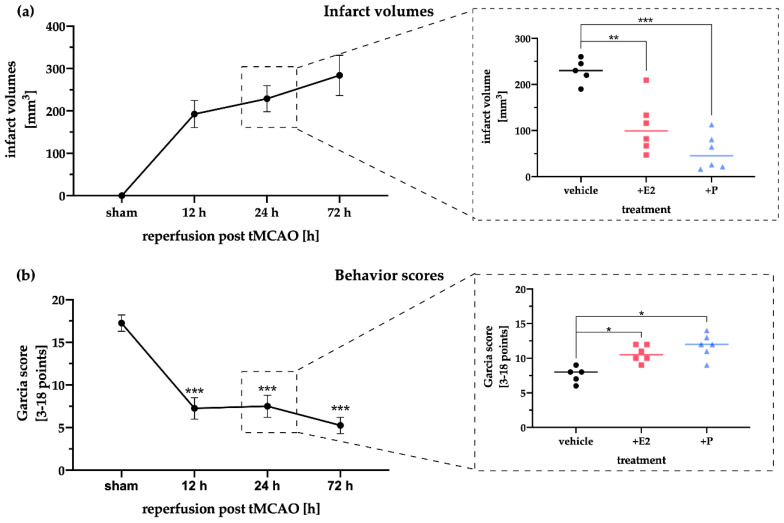
Determination of the infarct volume and the Garcia behavioral score after tMCAO. (**a**) The infarct volume increased over time with prolonged reperfusion and peaked at 72 h. Steroid hormone therapy with E2 or P significantly reduced the infarct volume at 24 h of reperfusion post tMCAO. Note that one animal in the E2 group exhibited a larger infarct volume compared to the other rats in the same group, but this value was not significant in the outlier test (Grub and Rout tests). (**b**). The behavior score dropped during reperfusion to a minimum at 72 h of reperfusion. After E2 and P therapy, higher Garcia scores were determined for both treatments. Note that a minimum score of 3, indicating maximum pathology, and a maximum of 18, no signs of any deficit, were possible. *p*-values: * ≤ 0.05; ** ≤ 0.01; *** ≤ 0.001, asterisks indicate sham vs time points (left panels) or vehicle vs E2/P treatment (right panels). Data represent the means ± SEM. *n* = 5 (sham) or *n* = 6 (E2/P). tMCAO–transient Middle Cerebral Artery occlusion.

**Figure A1:**
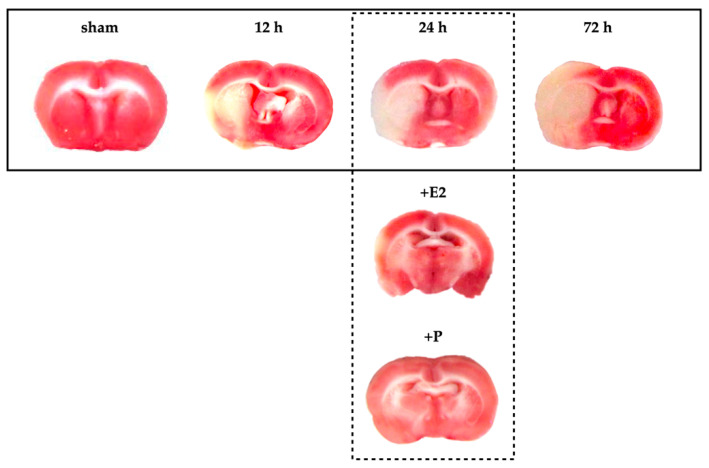
TTC-stained sections of brain slices. Brain slices were stained with TTC to discriminate living (deep red) from dead (unstained white areas) brain tissue. The infarct size increased with prolonged reperfusion time after tMCAO. E2 or P therapy significantly reduced the infarct size.

To study the influence of the gonadal steroid hormones E2 and P on post-ischemic brain damage and in particular on the regulation of the AIM2 and NLRC4 inflammasomes, we subjected a total of 65 male rats at the age of 12 weeks to a 1-hour tMCAO or sham surgery with a subsequent reperfusion time of 72 h. The primary endpoint of our study was the evaluation of infarct sizes and the resulting clinical impairment 12, 24, and 72 h after stroke. Here, a clear progression of infarct sizes with increasing reperfusion time was evident, whereby the largest lesion sizes were detected 72 h after tMCAO (Figure 1a). The administration of the steroid hormones E2 or P significantly reduced infarct sizes after 24 h of reperfusion compared to the corresponding vehicle controls. The latter is in line with previously published data [8]. Representative images of the TTC-stained brain sections demonstrating the infarct sizes of each treatment group and time-point are shown in Figure A1.

Neurological testing using the Garcia score revealed that the sham animals showed no deficits in performing the different tasks over the entire observation period of 72 h (Figure 1b). After tMCAO, performance deteriorated with increasing infarction volumes. Already after 12 h a considerable decline in performance was observed, which continued to decrease till the end of 72 h of reperfusion. E2 or P administration at 24 h post-stroke exhibited a beneficial improvement in the performance of the animals.

### 2.2. Gene and Protein Levels of the Inflammasomes AIM2 and NLRC4 Time-Dependently Increase after tMCAO

**Figure 2:**
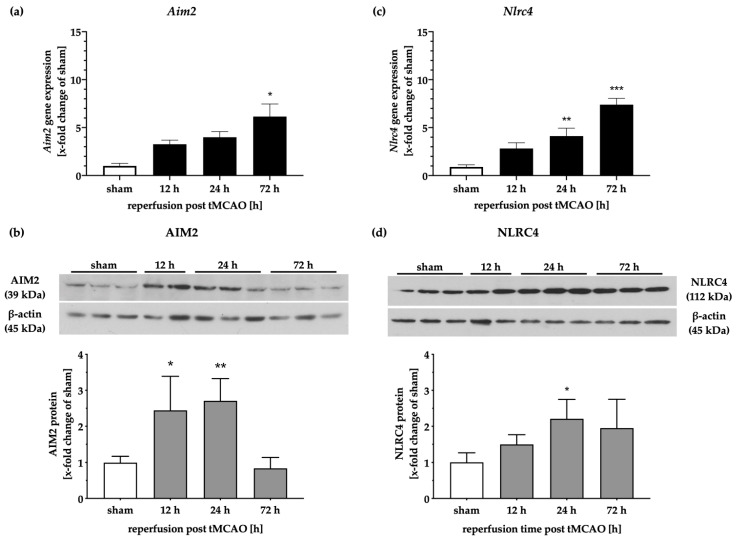
AIM2 and NLRC4 inflammasomes are increased in the peri-infarct brain tissue of rats. *Aim2* gene expression showed a steady increase after cerebral ischemia (**a**), whereas the protein levels displayed an initial increase after 12 and 24 h of reperfusion and decreased over the observation period of 72 h (**b**). The *Nlrc4* mRNA levels were elevated with increasing reperfusion time (**c**). Protein levels of NLRC4 were significantly increased after 24 h, followed by a slight decrease at the later reperfusion time point (**d**). *p*-values: * ≤ 0.05; ** ≤ 0.01; *** ≤ 0.001; asterisks indicate sham vs. time points. Data represent the means ± SEM. *n* = 5 (gene expression) or *n* = 4 (WB data). tMCAO–transient Middle Cerebral Artery occlusion.

To examine the expression patterns of AIM2 and NLRC4 on gene and protein levels in a time-dependent manner, we analyzed fresh tissue samples from biopsies taken post-mortem (Figure 2). While the *Aim2* transcripts increased with progressing reperfusion times and peaked after 72 h, the protein levels of AIM2 were elevated up to 24 h after tMCAO and decreased after 72 h (Figure 2a,b). After a reperfusion time of 72 h, AIM2 protein levels appeared to be identical to the sham group. Similar time-dependent increases of *Nlrc4* mRNA levels were observed following tMCAO (Figure 2c). However, the protein levels of NLRC4 exhibited a more moderate increase compared to AIM2 and decreased slightly over time (Figure 2d).

### 2.3. Steroid Hormones E2 or P Selectively Mitigate the Stroke-Induced Increase of AIM2 and NLRC4 Inflammasomes

**Figure 3:**
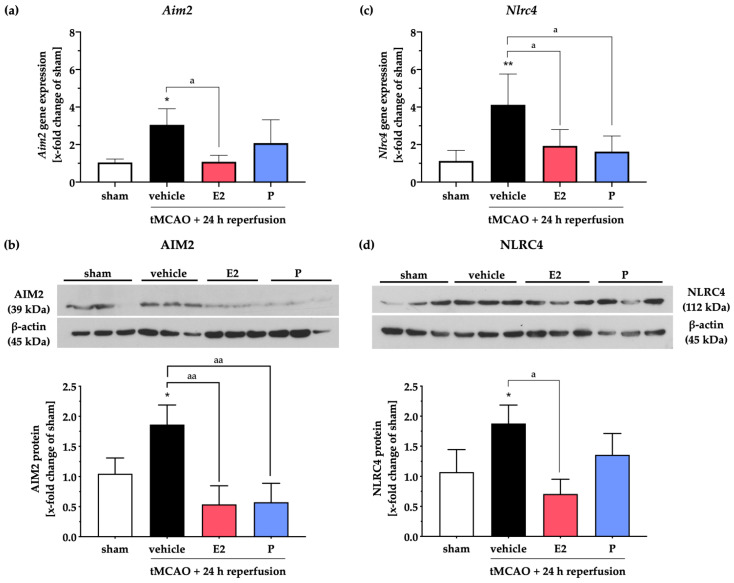
E2 or P therapy selectively modulates AIM2 and NLRC4 inflammasomes 24 h after cerebral ischemia. The AIM2 gene, as well as the protein levels, were elevated after tMCAO. The mRNA transcripts of Aim2 were significantly reduced after E2 therapy (**a**), whereas both steroid hormones were efficacious in AIM2 protein reduction in the peri-infarct area (**b**). Stroke exhibited a marked increase in NLRC4 gene and protein levels. Both steroid hormones were capable of diminishing Nlrc4 gene expression (**c**), but only E2 could reduce NLRC4 protein levels (**d**). *p*-values: */a ≤ 0.05; **/aa ≤ 0.01; asterisks indicate sham vs. the other groups; letters indicate vehicle vs. E2 or P. Data represent the means ± SEM. *n* = 5 (gene expression) or *n* = 4 (WB data). tMCAO–transient Middle Cerebral Artery occlusion; E2–17β-Estradiol; P–Progesterone.

Next, we analyzed the impact of E2 or P treatment on the gene and protein expressions of the aforementioned inflammasomes AIM2 and NLRC4 after tMCAO (Figure 3). In line with our findings above, post-ischemic *Aim2* mRNA levels were increased in the vehicle group after 24 h of reperfusion (Figure 3a). E2 administration prevented the post-stroke increase of *Aim2* mRNA, wherein P failed to induce a significant reduction of the *Aim2* transcript levels. However, the ischemia-induced increase of AIM2 protein levels was significantly attenuated by both gonadal hormones (Figure 3b).

Likewise, NLRC4 was elevated in both gene and protein levels in the vehicle group (Figure 3c,d). In contrast to their effects on *Aim2* transcripts, E2 and P administration dampened *Nlrc4* gene expression (Figure 3c). However, E2 but not P significantly reduced the stroke-dependent enhancement of the NLRC4 protein (Figure 3d).

**Figure 4:**
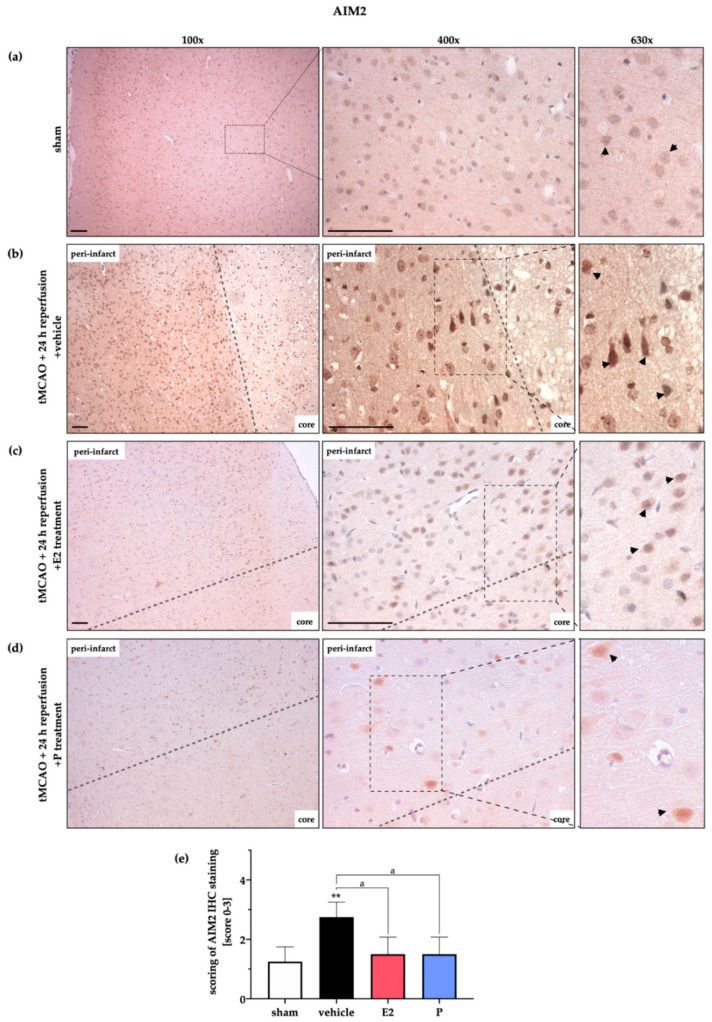
Evaluation of AIM2 protein expression in the peri-infarct zone after 24 h reperfusion by IHC. IHC staining of AIM2 in the peri-infarct area was utilized to strengthen WB data. (**a**) AIM2 displayed a low abundance in the cortex of the left hemisphere. (**b**) Post-ischemic brain tissue exhibited an increased AIM2 signal in cortical cells. The shape of the latter indicates that neurons most abundantly expressed AIM2 (black arrows under 630× magnification) (**c**) E2 therapy reduced the signals of the AIM2 staining around the ischemic core (core). Overall, the staining/background ratio seemed lower in comparison to the vehicle group (arrows under 630× magnification). (**d**) A similar reduction of AIM2 was detected after P administration. (**e**) Semi-quantitative evaluation by blinded scoring underlines the first impressions of increased AIM2 signals in the vehicle group and lower signals after E2 or P treatment. Scoring was evaluated by 1 = slight signal, 2 = medium signal, and 3 = strong signal. *p*-values: a ≤ 0.05; ** ≤ 0.01; asterisks indicate sham vs. the other groups; letter indicate vehicle vs. E2 or P. Data represent the means ± SEM. *n* = 3 (sham) or *n* = 4 (other groups). Black bars indicate a scale of 100 µm. The dashed line delineates the transition zone of the peri-infarct area to the ischemic core. tMCAO–transient Middle Cerebral Artery occlusion; E2–17β-Estradiol; P–Progesterone.

**Figure 5:**
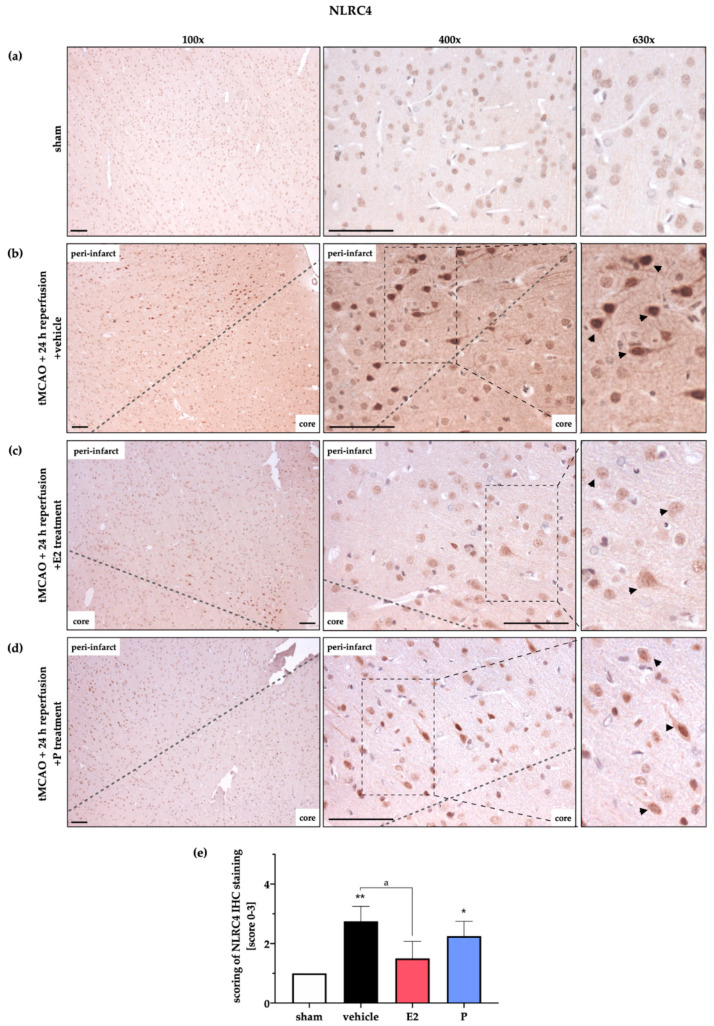
Evaluation of NLRC4 protein expression in the peri-infarct zone after 24 h reperfusion by IHC. IHC staining of NLRC4 in the peri-infarct area and the corresponding control area was used to validate and strengthen the WB data in Figure 3. (**a**) In sham animals, only a weak NLRC4 signal was detected. (**b**) The vehicle group revealed dense, deeply stained cells, indicating high NLRC4 expression in the transition zone after stroke (black arrows under 630× magnification). (**c**) E2 therapy reduced the NLRC4 signals, whereas P supplementation (**d**) showed a weak effect on NLRC4 expression. (**e**) Semi-quantitative evaluation by blinded scoring underlines the first impressions of increased NLRC4 signals in the vehicle group and lower signals after E2 but not P administration. Scoring was evaluated by 1 = slight signal, 2 = medium signal, and 3 = strong signal. *p*-values: */a ≤ 0.05; ** ≤ 0.01; asterisks indicate sham vs. the other groups; letter indicate vehicle vs. E2 or P. Data represent the means ± SEM. *n* = 3 (sham) or *n* = 4 (other groups). Black bars indicate a scale of 100 µm. The dashed line delineates the transition zone of the peri-infarct area around the ischemic core. tMCAO–transient Middle Cerebral Artery occlusion; E2–17β-Estradiol; P–Progesterone.

**Figure A2:**
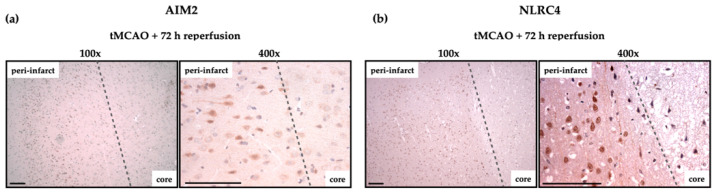
AIM2 and NLRC4 IHC staining of the late time point of 72 h reperfusion.

For validation and strengthening of our WB data, we performed IHC staining for AIM2 (Figure 4) and NLRC4 (Figure 5) after tMCAO or sham surgery followed by various reperfusion time points. We found weak to barely noticeable AIM2 signals in sham animals (Figure 4a), whereas intense staining of AIM2 was detectable in the peri-infarct area around the ischemic core of the vehicle-treated group (Figure 4b). Note that the pyramidal-shaped cells directly located next to the ischemic core and delineated by the grey dashed line in the representative images expressed high amounts of AIM2. At the end of the reperfusion period (72 h), AIM2 signals were comparable to sham animals, as also indicated in the WB data above (Figure A2a). The administration of E2, as well as P, displayed a marked reduction of AIM2 proteins in the peri-infarct area at 24 h of reperfusion (Figure 4c,d).

Analogous to AIM2, NLRC4 displayed a low abundance in sham animals (Figure 5a). Stroke evoked stronger DAB signals in the vehicle group after 24 h of reperfusion, indicating higher NLRC4 amounts (Figure 5b). The latter was mitigated by E2 administration, but not by P treatment (Figure 5c,d). In contrast to AIM2, NLRC4 signals seemed to remain increased after 72 h of reperfusion (Figure A2b). Note that the administration of P led to lower signals of NLRC4 in the cortex, but not in the peri-infarct area directly surrounding the ischemic core. In summary, gene expression analyses and WB and IHC staining indicate post-ischemic upregulation of the AIM2 and NLRC4 inflammasomes, which were counteracted by the steroid hormones E2 and P in a distinct manner.

### 2.4. AIM2 and NLRC4 Inflammasomes Seem to Be Most Abundant in Neurons Under Physiological and Ischemic Conditions

**Figure 6:**
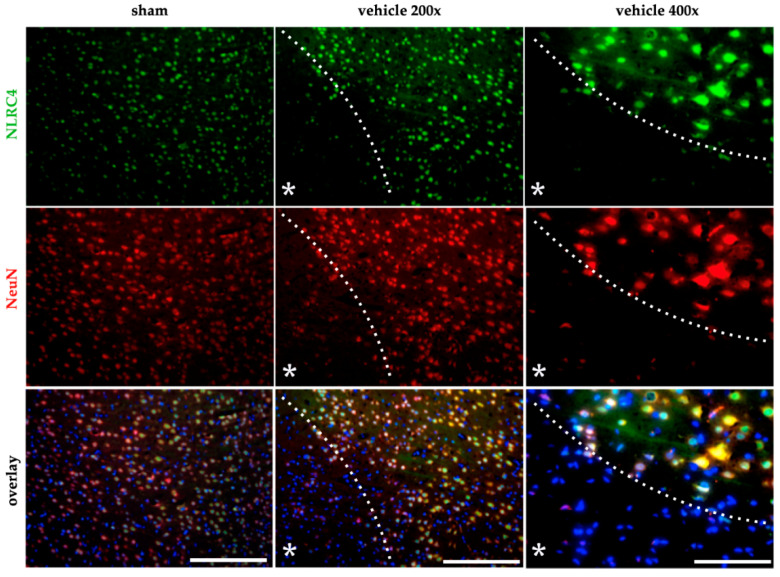
NLRC4 is mainly abundant in neurons after cerebral ischemia in rats. The co-localization of NLRC4 was evaluated by IF-co-staining with neurons (NeuN) in the sham and vehicle group (tMCAO + 24 h reperfusion). NLRC4 was mainly localized in NeuN + neurons in sham animals, as well as in the vehicle group after cerebral ischemia. White bars indicate a scale of 250 µm (200× magnification of sham and vehicle) or 100 µm (400× magnification). The dashed line delineates the transition zone of the peri-infarct area around the ischemic core (asterisk). NeuN–Fox-3, Rbfox3, or Hexaribonucleotide Binding Protein-3.

The IHC staining suggested, from a morphological point of view, that neurons predominantly seem to be the main source of the inflammasomes mentioned above. To clarify this, we investigated the expression of AIM2 and NLRC4 in neurons as well as in glial cells using fluorescence staining in normoxic and post-ischemic brains of rats. Despite numerous attempts, we were not able to establish the antibody against AIM2 in fluorescence staining, which worked in IHC. NLRC4, in turn, was exclusively detectable in NeuN-positive neurons (Figure 6) in sham animals, as well as in the vehicle-treated animals after tMCAO. Note that NLRC4 stained in NeuN-positive neurons delineated the infarct core from the peri-infarct zone.

**Figure 7:**
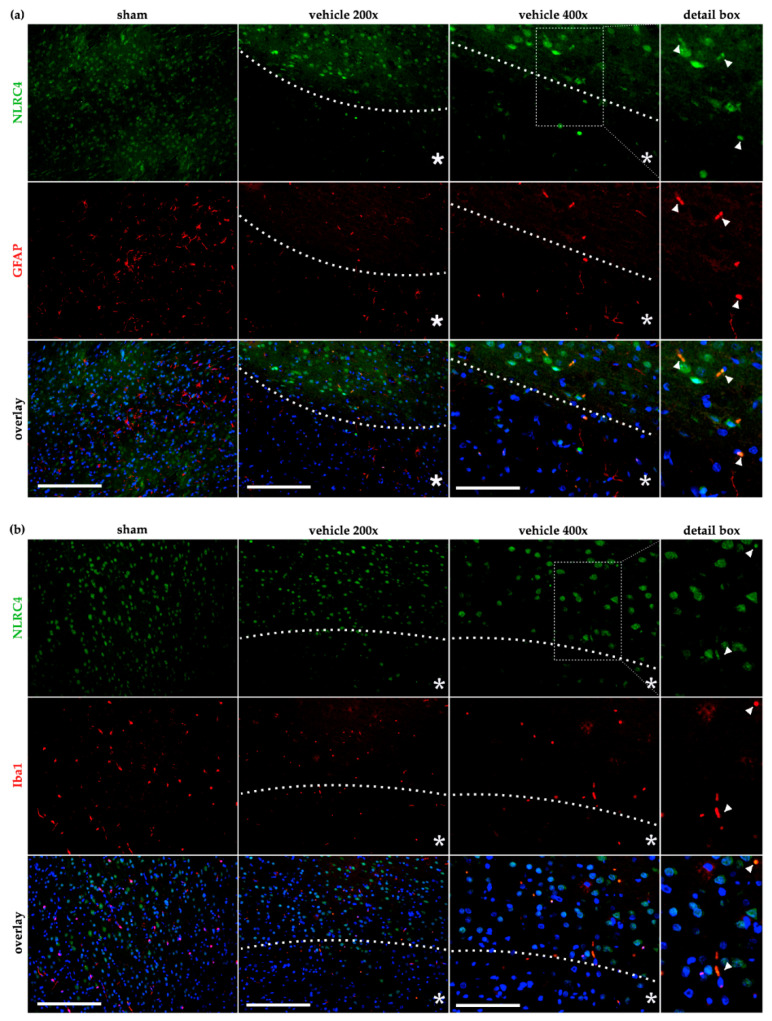
NLRC4 is occasionally detected in astro- and microglia cells after cerebral ischemia in rats. The co-localization of NLRC4 was evaluated by IF-co-staining in the sham and vehicle group (tMCAO+24 h reperfusion) with astrocytes (GFAP) (**a**) and microglial cells (Iba1) (**b**). (**a**) In astrocytes, low co-localization was evident in sham animals but occasionally appeared in the peri-infarct area in the vehicle group (see 400× magnification and the detail box). (**b**) For Iba-1 stained microglia, a similar result was observed that after cerebral ischemia, a slight amount of microglia seemed to co-localized with NLRC4 (see arrows in the detail box). Note that for proper visualization, exposure was adapted to show the co-localization between GFAP or Iba-1 positive cells with NLRC4. White bars indicate a scale of 250 µm (200× magnification of sham and vehicle) or 100 µm (400× magnification). The dashed line delineates the transition zone of the peri-infarct area around the ischemic core (asterisk). GFAP–glial fibrillary acidic protein; Iba1–ionized calcium-binding adapter molecule 1.

In contrast, double staining of both astrocytes (Figure 7a) as well as microglial cells (Figure 7b) with NLRC4 displayed no co-localization in sham animals. After cerebral ischemia in the vehicle group 24 h post tMCAO, sporadic co-localization was found in both cell types, indicating that a low number of astrocytes or microglia is capable of expressing NLRC4 after an ischemic insult. Nevertheless, our results demonstrate a predominant expression of NLRC4 in neurons under normoxic and ischemic conditions. To further investigate and validate our in vivo findings, we examined microglial cells and astrocytes in an in vitro ischemia model with respect to AIM2 and NLRC4 expression.

### 2.5. Administration of E and P Selectively Reduced AIM2 and NLRC4 in Primary Cortical Astrocytes and Microglial Cells after Oxygen–Glucose Deprivation

**Figure 8:**
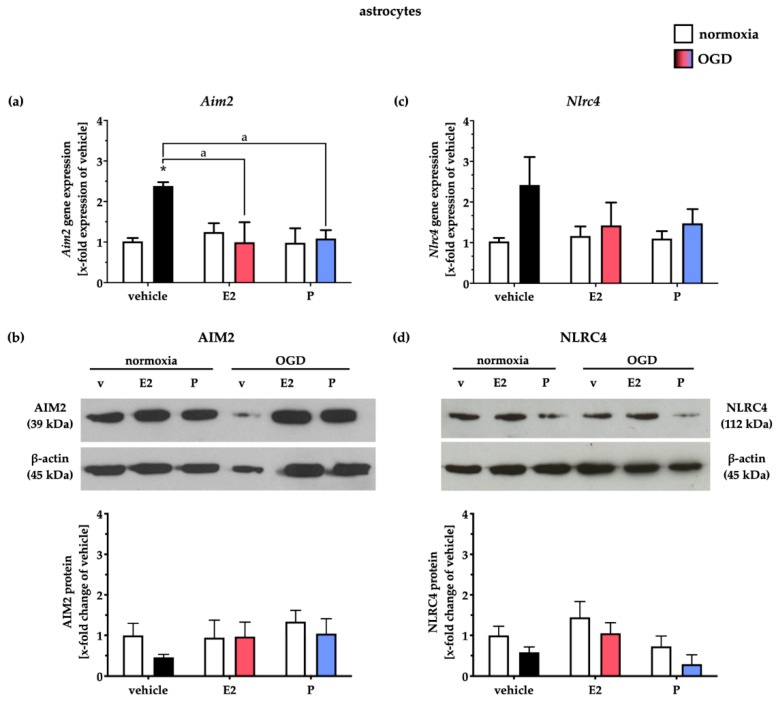
E2 and P treatment mitigated OGD-induced upregulation of *Aim2* mRNA levels in astrocytes. (**a**) *Aim2* gene expression was elevated after 3 h of OGD. Both steroid hormones abolished this effect. (**b**) OGD, as well as steroid hormones, seemed to not influence AIM2 protein levels in primary astrocytes. (**c**) *Nlrc4* mRNA, as well as NLRC4 proteins in astrocytes, did not appear to be influenced by OGD or the steroid hormones E2 and P (**d**). *p*-values: */a ≤ 0.05; asterisks indicate normoxia vehicle vs. other groups, letters indicate OGD vehicle vs. OGD + E2/P treatment. Data represent the means ± SEM. *n* = 4 per group. OGD–oxygen–glucose-deprivation; E2–17β-Estradiol; P–Progesterone.

Due to the lack of co-localization of AIM2 or NLRC4 inflammasomes in glial cells after tMCAO, we intended to investigate in single-cell cultures (microglia and astrocytes) whether these inflammasomes are present and whether their regulation is influenced by oxygen–glucose deprivation. Furthermore, in the case of glial expression of both inflammasomes, we aimed to investigate the influence of E2 and P treatment on the regulation of the aforementioned inflammasomes after in vitro ischemia. OGD significantly enhanced the mRNA transcripts of *Aim2* in primary astrocytes, which was mitigated by E2 and P supplementation (Figure 8a). Interestingly, we detected a slight but not statistically significant reduction of the AIM2 protein levels after OGD, which, however, was subsequently increased to the levels of the normoxia control after the administration of both steroid hormones (Figure 8b). NLRC4 inflammasomes in astrocytes did not appear to be influenced by OGD or the steroid hormones E2 and P, indicating a minor role of NLRC4 in primary cortical astrocytes after in vitro ischemia (Figure 8c,d).

**Figure 9:**
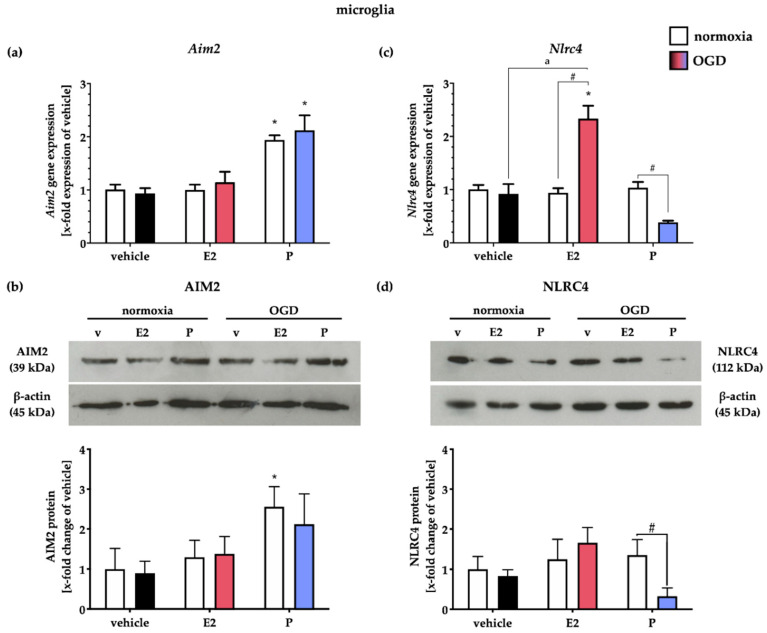
E2 and P treatment appear to selectively influence NLRC4 gene and protein expression in primary cortical microglial cells from rats after OGD. (**a**) OGD did not impact *Aim2* mRNA expression, whereas P increased it already in normoxic controls. (**b**) At the protein level, AIM2 levels were slightly increased after P treatment under normoxic as well as hypoxic conditions. (**c**) *Nlrc4* gene expression was either increased after E2 treatment or reduced after P treatment in combination with OGD, while no effect of OGD was evident. (**d**) Interestingly, selective treatment with P after OGD reduced NLRC4 protein levels. *p*-values: */a/# ≤ 0.05; asterisks indicate normoxia vehicle vs. other groups, letters indicate OGD vehicle vs. OGD + E2/P treatment, hashtags indicate normoxia vs. OGD per group. Data represent the means ± SEM. *n* = 4 per group. OGD–oxygen-glucose-deprivation; E2–17β-Estradiol; P–Progesterone.

Primary microglial cells exhibited a basal *Aim2* inflammasome expression at both gene and protein levels under normoxic conditions (Figure 9a,b), which underlines our in vivo data, that microglia cells are capable of expressing AIM2 in the rodent CNS. Interestingly, P treatment increased *Aim2* gene expression under normoxic conditions.

OGD did not regulate *Aim2* gene expression; neither did the gonadal steroids (Figure 9a). AIM2 protein levels were similar to the gene level increase only after P supplementation in normoxia controls. Here, OGD did not further alter AIM2 protein levels (Figure 9b).

The *Nlrc4* gene expression was unchanged after OGD or steroid hormone treatment in normoxia controls. OGD, in combination with E2, increased *Nlrc4* expression, whereas P decreased it (Figure 9c). Similar to AIM2, the NLRC4 protein levels were neither affected by OGD nor by E2 supplementation. Administration of P was capable of decreasing NLRC4 protein levels after OGD (Figure 9d).

## 3. Discussion

The current study provides evidence that, besides NLRP3, the most prominent member of the inflammasome family, the less described AIM2 and NLRC4 inflammasomes, are also upregulated after ischemic stroke and are affected in their expression by the protective gonadal steroids E2 and P. We were able to demonstrate that the administration of E2 and P decreased both infarct sizes and neurological impairments after cerebral ischemia in rats. Furthermore, we observed a time-dependent elevation of gene and protein levels of the AIM2 and NLRC4 inflammasomes in the post-ischemic brains. In addition to the reduction of the infarct volumes described above, E2 or P selectively mitigated stroke-induced increase of AIM2 and NLRC4. While both inflammasomes seemed to be exclusively abundant in neurons under physiological and ischemic conditions in vivo, single-cell cultures of cortical astrocytes and microglia equally expressed both inflammasomes. In line with the in vivo data, E and P selectively reduced AIM2 and NLRC4 in primary cortical astrocytes and microglial cells after oxygen–glucose deprivation.

The protective effect of E2 and P after ischemic stroke in rats in this work confirms and reinforces previous studies [8,20,21]. The aforementioned studies also observed the most potent protective effects after hormone application 24 h after stroke (Figure 1). However, due to the reperfusion time investigated in this study, the question might arise whether the hormone application after stroke only leads to an initial slowing of the infarct progression, which might result in an infarct volume similar to that of the vehicle-treated animals over time. The long-term effect of both gonadal hormones after stroke has already been investigated by our group and revealed that hormone administration in the initial phase after tMCAO (24 h) has a prolonged protective effect by significantly reducing not only the infarct volumes but also the neurological impairments (Rotarod, Garcia score) over 14 days after stroke [22]. Hence, a prolonged gonadal hormone-mediated cytoprotection can be assumed, even if the hormone administration was only carried out in the initial phase after cerebral ischemia. Lammerding et al. reported an upregulation of *Aim2* and *Nlrc4* mRNA levels in the post-ischemic brain, but interestingly, mRNA levels of *Nlrp3* seemed to be downregulated in neurons [8], while other studies, in contrast, consistently described an upregulation of NLRP3 in the peri-infarct zone or in the neurovascular unit [23]. These controversial findings may be since upregulation of NLRP3 has been observed in mice rather than in rats [6,7]. While many studies propagate the inhibition of NLRP3, for example, by small molecule inhibitors such as MCC950, as a protective strategy after stroke [24,25], other data suggest a marginal role of NLRP3 after ischemic stroke. In 2015, Denes and colleagues were able to illustrate that AIM2 and NLRC4 inflammasomes seem to have a significantly more substantial influence on infarct volumes and behavior than NLRP3 [9]. In line with the latter, a recently published study supports that NLRP3 inhibition and knockout seem not to influence stroke sizes after tMCAO, indicating that the NLRP3 pathway does not contribute to the inflammation exacerbating ischemic brain damage [26]. Besides, we have recently demonstrated that NLRP3 depletion in the murine microglial cell line BV-2 does not lead to an altered inflammatory cascade, but the hypoxia-driven reduction of phagocytosis is restored [27]. More recent studies from 2020 suggest that modulation of AIM2 leads to lower infarct volumes and reduced activation of the inflammatory cascade. Kim and colleagues described that the AIM2 inflammasome contributes to brain injury and chronic post-stroke cognitive impairment in mice [28]. Furthermore, it has been shown that the cyclic GMP-AMP synthase antagonist A151 effectively reduced the expression of AIM2 and its downstream cascade, thereby significantly reducing neuro-deficits and diminished cell death [29]. Moreover, Liang and colleagues have indicated AIM2 as a direct target of miR-485 and postulated that the long non-coding RNA MEG3 promotes cerebral ischemia-reperfusion injury by increasing pyroptosis by targeting the miR-485/AIM2 axis [30].

In the case of NLRC4, there are limited data regarding its role after a stroke. Poh and colleagues provided evidence in 2019 that NLRC4 inflammasomes mediate both the inflammatory response, as well as apoptotic and pyroptotic cell death in murine microglial cells subjected to in vitro and in vivo ischemia [31]. NLRC4 also seems to play a role in astrocytes after ischemia. Sui and colleagues were able to point out that both NLRP3 and NLRC4 were markedly increased in TNA2 astrocytes from rats exposed to OGD [32]. However, in the study mentioned above by Kim et al., the authors observed that AIM2 mRNA and protein increased until seven days post-stroke with an AIM2 immuno-reactivity primarily co-localized in microglial (Iba-1) and endothelial cells (CD31). However, immuno-reactivities neither in neurons (NeuN) nor in astrocytes (GFAP) were detected, suggesting that microglial or endothelial cell-induced AIM2 production mediated post-stroke cognitive impairment pathogenesis.

In line with the above, we observed weak signals of immuno-reactivity of AIM2 and NLRC4 in astroglial and microglial cells. Still, both inflammasomes were found most abundantly in neurons in the post-ischemic brain (Figure 7). In primary cortical glial single-cell cultures (microglia or astrocytes) from rats, we were able to detect the expression of AIM2 and NLRC4 at both gene and protein levels. OGD for 3 h, however, had no impact on AIM2 and NLRC4 protein levels (Figure 8 and Figure 9). The different reports on the cellular distribution of inflammasomes after ischemia are, in our opinion, due to the different species (mice or rats) used, but probably also to a large extent due to the temporal evaluation of the expression of inflammasomes after stroke. While the studies mentioned above in part allowed reperfusion 7 days after stroke, we investigated a maximum reperfusion time of 72 h in vivo and analyzed the expression of the inflammasome directly after a 3-h OGD in in vitro. This can be seen as a limitation of the study because a clear statement on the expression of the inflammasomes at the cellular level over time would be more valid. This also applies to infarct progression and its reduction by the steroid hormones E2 and P. The fact that our data from the micro- and astroglial cell cultures after OGD do not fully reflect the in vivo data is partly due to the circumstance that we studied the interaction of only two cell types in cell cultures and neglected the impact of neurons, oligodendrocytes, pericytes, endothelial cells, and migrated immuno-competent cells in the post-ischemic phase. Although an OGD mimics certain features of cerebral ischemia, factors such as increased intracranial pressure and blood perfusion, among others, cannot be adequately depicted in vitro. Furthermore, it is essential to note that the cortical cells we used were prepared from postnatal (p0-p2) cells. Thus, we did not have the same degree of maturity as in our in vivo cells. Due to these limitations, the translational applicability of OGD data to the in vivo situation is limited. A further limiting aspect, which we partly share with the studies mentioned above, is the assessment of the expression but not the activation of the inflammasomes after stroke. Future studies will need to investigate the activation of inflammasomes and their tempero–spatial expression after stroke using different occlusion times of MCA and more extended reperfusion periods.

The assumption that the steroid hormones E2 and P have a neuroprotective effect after experimental stroke is based on a large number of studies [8,11,13,14,15,19]. Also, their modulating influence on the post-ischemic inflammatory cascade and especially their attenuating influence on IL-1β and IL18 are well documented [19,33,34,35]. An increasing amount of literature has been accumulated on the role of inflammasomes after ischemia/hypoxia in the central nervous system [36,37,38,39,40]. Yet, the mechanisms of action of E2 and P on the inflammatory cascade and especially on the modulation of inflammasomes are poorly understood. We demonstrate that AIM2 and NLRC4 are upregulated after ischemia and E2 + P seem to downregulate AIM2, while NLRC4 seems to be lowered only by E2. These results could be due to the different classical and non-classical E2 and P receptors, whose downstream function is not fully understood. Depending on which receptor the hormones bind to, different cascades with different kinetics can be initiated [41]. Although there is little literature on the estrogen receptors (ERalpha, ERbeta, and GPR30) for cancer and in connection with NLRP3, their impact on NLRC4 and AIM2 is not described yet [42,43]. In addition, the concentration and temporal administration of both hormones might also influence the detected effects. Based on our previous studies and studies of other groups, we know a concentration range for the two hormones, where we do not expect toxicity but protective effects [20,22]. However, it is not clear whether higher or lower concentrations within the protective concentration range might have a different impact on the inflammasomes. Future studies will have to address not only the temporal aspect but also the different concentrations and use selective inhibition of steroid receptors to investigate the underlying mechanisms of steroid hormone-dependent regulation of AIM2 and NLRC4 inflammasomes after ischemia.

Under in vitro conditions, three hours of OGD were sufficient to induce an increase of *Aim2* and *Nlrc4* mRNAs, which was counteracted by both steroid hormones. Whether this also occurs at the protein level probably requires different OGD and reperfusion times, which will be investigated in future studies. Overall, there is little literature on treatment with E2 or P in glial cells, especially with regard to their impact on inflammasomes.

Based on our findings and previous reports, we provide evidence that the neuroprotective/anti-inflammatory effects of E2 and P are at least partly mediated through the modulation of inflammasomes. Besides NLRP3, AIM2 and NLRC4 seem to play a crucial role after ischemia and are regulated by E2 and P.

## 4. Materials and Methods

### 4.1. Animals and Animal Care

**Figure 10:**
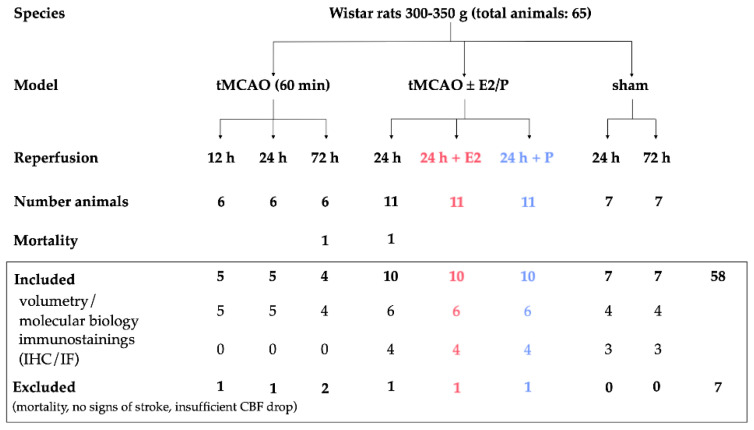
Overview of animal numbers used in the study and allocation to experimental groups. Note that for volumetric evaluation of infarct volumes and molecular analysis, we utilized the same animals per group, except for one animal being excluded for molecular analysis due to a particular large infarct size compared to the other rats in the group. For IHC and IF, we used the same animals for the sham and 24-h vehicle. tMCAO–transient Middle Cerebral Artery occlusion; E2–17β-Estradiol; P–Progesterone

All experiments and procedures, including animals, were approved by the Review Board for the Care of Animal Subjects of the distinct government (North Rhine-Westphalia, Germany, 84-02.04.2013.A212, approved January 2014). All animal experiments were performed on 12-week-old male Wistar rats (Charles-River, Sulzfeld, Germany) with a weight range of 300–350 g. To avoid the effect of endogenous E2 and P and to ensure comparability with previous studies, we have been using male Wistar rats for years, since ovariectomized rats still exhibit higher levels of gonadal steroids than male rats [20]. In total, 58 Wistar rats were included in this study. Seven animals were excluded due to the absence of stroke symptoms or unexpected death before the end of the experiment. An overview of the total included animals and allocation to the experimental groups is summarized in Figure 10. Animals were identified by earmark numbers and were randomly assigned to the treatment groups by a technical assistant not involved in the analyses. Randomization was carried out through sorting by random numbers (QuickCalcs, Graphpad Prism, version 6.0 for Mac, GraphPad Software, San Diego, California USA, www.graphpad.com). Based on our preliminary data, in which we observed a reduction of infarct volumes of at least 40 to 70% after gonadal hormone application, we consider 20% differences in infarct sizes as significant in this study. Therefore, we required a minimum of three animals per group to detect such a difference at a 95% confidence level (a = 0.05) and 0.8 power. Power analysis was carried out with G*Power. A test for outliers was not conducted on the data. The individual numbers of animals in our in vivo experiments are shown in the figure legends. Rats were bred and maintained in a pathogen-free environment with a maximum of five animals per cage under a 12-h day and 12-h night cycle. Food and water were available *ad libidum*. Once a week, animals underwent routine cage maintenance, according to the Institute for Laboratory Animal Science and Experimental Surgery (University Hospital Aachen, RWTH Aachen University) guidelines and microbiological monitoring, according to the Federation of European Laboratory Animal Science Association recommendations. All experiments involving animals are reported in accordance with the ARRIVE guidelines for reporting experiments involving animals [44].

### 4.2. Stroke Surgery (Transient Middle Cerebral Artery Occlusion, tMCAO)

The procedure of transient middle cerebral artery occlusion (tMCAO) was performed as previously described [8,21]. Briefly, rats were anesthetized with 2–3% isoflurane (Abbott, Wiesbaden, Germany), and the regional cerebral blood flow (CBF) of the ipsi- and contralateral hemisphere was monitored using Laser Doppler flowmetry (Moor Instruments VMS-LD2, Millwey, UK) during the whole surgical procedure. During the operation, body temperature was kept constant at 37.0 ± 0.5 °C. After exposure of the *bifurcatio* of the common carotid artery (CCA), external carotid artery (ECA), and internal carotid artery (ICA), the CCA was ligated at the proximal side. After insertion of a commercially available silicon-coated filament (Doccol Corporation, Sharon, Massachusetts, USA) into the CCA, it was pushed forward to occlude the MCA branch, which was paralleled by a baseline drop in the CBF. Sham animals underwent the same procedure, except the filament was not threaded forward to occlude the MCA. Animals with a decline of the CBS of 50% or less were excluded from the study. After 1 h of MCA occlusion, the filament was removed, and the CBF was restored. After tMCAO, animals were kept under a heating lamp until they finally woke up. Animals were randomly assigned to the experimental groups and were allowed to recover for 6, 12, 24, and 72 h for the time course study and 24 h for the steroid hormone treatment.

### 4.3. Behavior Testing

For the evaluation of effective stroke surgery, we assessed neurological deficits by motor and sensory behavioral tasks with slight modifications [45]. The behavioral testing was performed by two blinded investigators to the experimental procedures before animals were sacrificed for sampling. The following six tests were performed: Spontaneous activity was analyzed for 3 min within an unfamiliar environment by placing the animals in the middle of a 35 cm × 55 cm sized cage. For testing forepaw outstretching, rats were fixed at the tail, and the symmetry of the outstretching of both forelimbs was evaluated. To assess the climbing ability, rats were placed on the wall of a wire cage. Usually, rats use all four limbs to climb the wall. For testing body proprioception, rats were touched with a blunt stick on each side of the body, and the reaction to the stimulus was evaluated. The spontaneous walking activity was staged. The sensory function was tested by brushing the vibrissae. All tests and the scores are summarized in Table 1. Individual scores of all tests were summed. An overall minimum score of 3 and maximum score of 18 points were achievable.

### 4.4. Hormone Application

Rats received E2 or P (Sigma Aldrich, Taufkirchen, Germany) dissolved in pure ethanol. The treatment with E2 or P was conducted in a randomized manner directly after the withdrawal of the filament. The application was repeated every 12 h until the final sacrifice of the animal. A mixture of E2 (25 µg/kg body weight) or P (10 mg/kg body weight) in sesame oil was given as a neck depot in a final volume of 500 µL. Sham animals received a neck depot of sesame oil and ethanol. Experimental bias was avoided that a person blinded to the tMCAO operation gave the content of the coded neck depot solution to the experimenter. After the experiment, the animal was sacrificed, and the brain removed. Afterward, the brain tissue was sliced and stained with TTC to outline the peri-infarct area for tissue sampling.

### 4.5. TTC Staining and Tissue Sampling

After the experiment, animals were transcardially perfused with ice-cold PBS. Brains were rapidly removed, and six consecutive coronal sections (2 mm) were prepared. Afterward, the sections were stained in 2% 2,3,5-triphenyltetrazolium chloride (TTC, Fluka, Buchs, Switzerland) solution for 15 min at 37 °C. The unstained areas were defined as the infarct area, which delineates the transition zone from infarct to non-infarct area. All further molecular and biochemical analyses were only performed with tissue extracted from TTC-stained brain sections that represented the transition zone. Tissue samples were taken with a 2 mm biopsy punch.

### 4.6. Primary Astroglia and Microglia Culture

Primary cortical astroglial and microglial cells were isolated as previously described with minor modifications [11,13]. Briefly, cerebral cortices from neonatal 1–2-d-old rat pups were carefully removed, minced, and dispersed in Dulbecco’s phosphate-buffered saline (PBS, ThermoFisher Scientific, Waltham, Massachusetts, USA) containing 1% trypsin and 0.02% EDTA (ThermoFisher Scientific, Waltham, Massachusetts, USA) for 10 min. After centrifugation at 400 g for 5 min (all centrifugation steps were made at room temperature, almost ~21 °C), the cell pellet was resuspended in Dulbecco’s modified Eagle medium (DMEM, ThermoFisher Scientific, Waltham, Massachusetts, USA), minced, and filtered through a 40 µm nylon mesh. Next, cells were centrifuged at 400 g for 5 min, resuspended in DMEM supplemented with 10% heat-inactivated fetal calf serum (FCS) and 0.5% penicillin/streptomycin (PS, all ThermoFisher Scientific, Waltham, Massachusetts, USA) and finally seeded on poly-L-ornithine (PLO) (Sigma Aldrich, Taufkirchen, Germany) coated culture flasks. After confluency (sealed cell layer), microglia were detached by a mild rotary shaking protocol at 130 rpm and 37 °C for 2 h followed by direct cell seeding in the culture medium on experimental well plates for one day. Astrocytes were separated from other cells by further shaking at an increased rotary speed of 230 rpm and 37 °C for 18 to 20 h. Subsequently, the astrocyte layer was detached and sub-cultured with medium renewal every three days. For experiments, cells were seeded in DMEM culture medium until the monolayer reached confluency. Afterward, the medium was exchanged with starving Roswell Park Memorial Institute (RPMI) medium 1640 without phenol red (ThermoFisher Scientific, Waltham, Massachusetts, USA) adjusted to 1 mg/mL glucose and supplemented with 5% hormone-free charcoal-stripped FCS (CSFCS, ThermoFisher Scientific, Waltham, Massachusetts, USA) and 0.5% PS 24 h prior to hypoxia induction. The microglia cells were seeded in culture plates in a density of 80,000 cells/cm^2^, whereas astrocytes were seeded on PLO-coated cell culture plates in a density of 65,000 cells/cm^2^ for gene and protein expression studies. The final microglia and astrocyte cultures were characterized by >95% homogeneity of Iba1 (ionized calcium-binding adapter molecule 1) or GFAP (glial fibrillary acidic protein)-positive cells as routinely performed in our laboratory and has been described several times [46].

### 4.7. Oxygen–Glucose Deprivation (OGD) and Steroid Hormone Treatment

To study the impact of oxygen–glucose deprivation (OGD) on the expression of NLRC4 and AIM2 in primary astrocytes and microglia cells isolated from rat pups, we utilized a cube-shaped hypoxia chamber, which was flooded with inert nitrogen gas with minor modifications to our previous reports [11,12,13]. Hypothermia was avoided by pre-warming the nitrogen gas using a water bath to 37 °C before injection into the chamber. Gas ventilation in the chamber was reached using a ventilation system. The oxygen levels were strictly controlled by an oxygen detector in the exhaust gas (Gox 100T, Greisinger Electronic GmbH, Regenstauf, Germany), and in the medium of experimental cell culture plates (FireStingO2, Pyro Science GmbH, Aachen, Germany).

**Figure 11:**
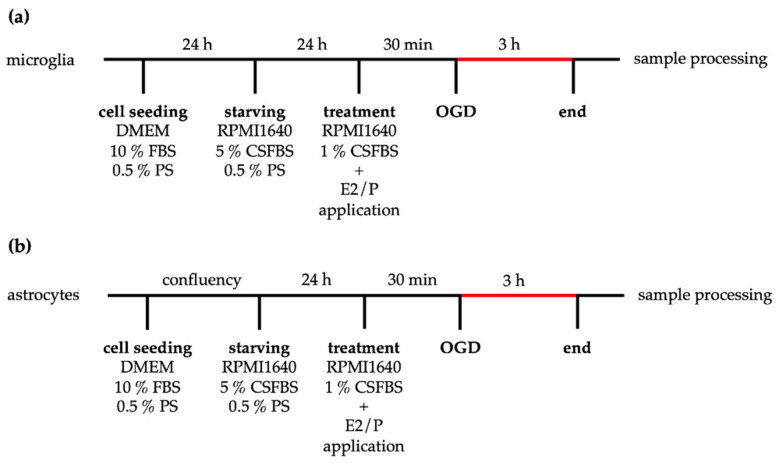
Scheme of the OGD experimental design. After isolation, microglia (**a**) or astrocytes (**b**) were seeded in the culture medium. Microglia were starved in RPMI 1640 5% CSFBS on the next day, whereas astrocytes (**b**) were maintained in the culture medium until the resulting monolayer stopped cell proliferation. Then, astrocytes were cultured with RPMI 1640 5% CSFBS for 24 h. Prior to OGD, cells received new RPMI1640 plus 1% CSFBS medium with or without E2 or P supplementation. After 3 h of OGD and the corresponding time under normoxic conditions, samples were further processed for analysis.

One day before OGD, the medium was changed for both cell types to a pre-treatment medium (RPMI 1640, 5% CSFBS, 0.5% PS). Note that for steroid hormone treatment, FCS was exchanged to steroid-free charcoal-stripped fetal bovine serum during pre- and hormone treatments. For hormone treatment, water-soluble and cell culture-tested E2 (E2758, Sigma Aldrich, Taufkirchen, Germany) and P (P8783, Sigma Aldrich, Taufkirchen, Germany) were diluted in pro analysis ethanol to yield a final concentration of 10^−7^ M in the treatment medium (RPMI 1640 1% CSFCS 0.5% PS). These concentrations correspond to 27.24 ng/mL (E2) and 31.45 ng/mL (P), respectively. Controls were treated with the same concentration of ethanol in the treatment medium. The cells were exposed to OGD for 3 h. Corresponding normoxia controls were kept under normal growth conditions with the same experimental media (RPMI 1% CSFCS, 0.5% PS). The experimental procedure of OGD is summarized in Figure 11.

### 4.8. RNA Extraction

For gene expression studies, the analysis was performed with the tissue of the peri-infarct region or cell lysates. Tissue samples were dissected using a stereomicroscopic approach, pooled, dissolved in peqGOLD Trifast^TM^ (VWR, Darmstadt, Germany), and homogenized. For cell culture samples, the supernatant was discarded, the cell layer was washed once with phosphate-buffered saline (PBS), and then peqGOLD Trifast^TM^ was placed on the cells. The extraction of total RNA was done following the manufacturers’ protocol, as previously described [47]. The RNA concentration and purity were measured with a NanoDrop 1000 device (VWR, Darmstadt, Germany).

### 4.9. Semi-Quantitative Real-Time PCR

Complementary DNA of 500 ng/mL total RNA was synthesized using the SensiFAST^TM^ cDNA Synthese kit (Bioline Meridian Bioscience, Cincinnati, Ohio, USA). Primers for mRNA analysis were designed with Primer-BLAST and are listed in Table 2 [48]. In 96-well plates (BIOplastics, Landgraaf, Netherlands), target and housekeeping genes were measured at the cycle threshold (Ct-values) by semi-quantitative reverse transcription PCR (qrt-PCR) in the CFXconnect qrt-PCR detection system (Bio-Rad, Hercules, California, USA). The PCR cycling conditions were as follows: 10 min denaturation at 95 °C, 40 cycles of 15 s denaturation at 95 °C, 30 s primer hybridization at the specific primer temperature, and 30 s annealing at 72 °C. By several-fold dilutions of target genes, external standard curves were generated in each run. After amplification, a melt curve analysis was additionally performed. To exclude mistakes and to check for unspecific products, agarose gel analysis was routinely performed. Using the qbase+ software (Biogazelle, Gent, Belgium), the relative quantification was calculated by the ΔΔCt-method, and data were expressed as the relative amount of the target to the amount of two or three housekeeping genes, namely, cyclophilin A (CycloA), and Glycerinaldehyd-3-phosphate-Dehydrogenase (Gapdh) for in vivo data, hypoxanthine phosphoribosyltransferase 1 (HPRT), β2-microglobulin (B2M), and TATA-binding protein (TBP) for in vitro data by using the multiple reference gene normalization method. Normoxia controls were set to 1.

### 4.10. Western Blot Analysis

Western Blot (WB) analysis was performed as previously described [14]. In brief, 20 µg of total protein per lane was separated by electrical voltage through SDS-PAGE. The proteins were transferred onto a PVDF membrane (Roche, Basel, Switzerland) and blocked with 5% dry milk in Tris-buffered saline containing 0.01% Tween (TBST). The primary antibody diluted in 5% blocking solution was incubated at 4 °C overnight on a rotary shaker. On the next day, the membranes were washed and incubated with horseradish peroxidase-conjugated secondary antibody. Labeled protein-antibody conjugates were visualized using the ECL™ Plus kit (ThermoFisher Scientific, Waltham, Massachusetts, USA). Beta-actin (β-actin) served as a loading control. We used Quantity One (Bio-Rad, Hercules, California, USA) to evaluate the resulting blotting bands. All bands were adjusted with their respective β-actin-bands. In Table 3, the used antibodies and their dilutions in this study are presented.

### 4.11. Immunohistochemistry

For immunohistochemistry, sections were dewaxed, rehydrated, and, if necessary, antigens were heat unmasked. Sections were blocked with 5% normal horse or goat serum in PBS and incubated at 4 °C overnight with the primary antibody diluted in blocking solution (see Table 3). After that, slides were first incubated with 0.3% H2O2 in PBS for 30 min in order to block the endogenous peroxidase followed by incubation with the secondary anti-rabbit or anti-mouse antibody diluted in blocking solution (see Table 3). Then, slices were incubated with peroxidase-coupled avidin-biotin-complex (VECTASTAIN Elite ABC Kit, Vector Labs, Burlingame, USA), which was visualized by 3,3′-Diaminobenzidine (DAB) working solution (Dako, Jena, Germany). Slides were counterstained with Mayer’s hematoxylin (Merck–Millipore, Darmstadt, Germany) for 30 s to visualize the cell nuclei. In a final step, all sections were dehydrated and mounted with Immu-Mount (ThermoFisher Scientific, Waltham, Massachusetts, USA).

For semi-quantification, IHC/IF staining were scored by blinded investigators. For this purpose, images were virtually blinded with VirtualBlind, in house-developed software. Afterwards, staining were scored using the following classifications: score 1 = slight DAB staining, score 2 = medium DAB staining, score 3 = strong DAB staining.

### 4.12. Immunofluorescence Stainings

Immunofluorescence double-labeling was performed as previously described [49]. Formalin-fixed and paraffin-embedded sections (5 m) were rehydrated, unmasked by Tris/EDTA pH 9.0 buffer, blocked in PBS containing 2% FCS, and 1% BSA, and incubated overnight with the primary antibodies diluted in blocking solution. A list of used antibodies is given in Table 3. The following antibodies, anti-glial fibrillary acidic protein antibody (GFAP), anti-neuronal nuclear antigen (NeuN), and ionized calcium-binding adapter molecule 1 (Iba1) were combined with NLRC4. Fluorescent anti-rabbit antibody (1:500; Alexa Fluor 488, Invitrogen, Germany) and anti-mouse/anti-goat antibody (1:500; Alexa Fluor 598, Invitrogen, Germany) were used as secondary antibodies. Hoechst 33342 was used for counterstaining the nuclei. Fluorescence images for qualitative expression analysis were acquired with a Leica DMI 6000 B.

### 4.13. Data Analysis

For statistics, 4–5 animals per group were used for molecular biological analysis and 3–4 animals per group for histological analysis. All in vitro experiments were performed in quadruplicate with 2–3 replicates per group. Residuals were analyzed for normal distribution using the Shapiro–Wilk normality test. For one-way ANOVA (all in vivo experiments), variance homogeneity was tested using the Bartlett test. For two-way ANOVA (all in vitro experiments), variance homogeneity was tested using Spearman’s rank correlation test for heteroscedasticity. When the normality or homogeneity test was significant, values were BOX-COX-transformed and reevaluated for normal distribution and variance homogeneity. Intergroup differences were tested by one-way ANOVA or two-way ANOVA, followed by Tukey’s posthoc test. All statistics were performed using GraphPad Prism version 8.3.1 for Mac, GraphPad Software, San Diego, California USA, www.graphpad.com. Data are given as arithmetic means ± SEM. The *p*-values are given as *p* ≤ 0.05, *p* ≤ 0.01, and *p* ≤ 0.001. Asterisks, letters, and hashtags indicate statistical differences. For details, see the figure legends.

## Figures and Tables

**Table 1:** Overview of the Garcia behavioral score.

Behavior Test	Points ^1^
Spontaneous activity	3 = rat moving around and exploring at least three walls 2 = rat moving around but not approaching the walls or eventually rising to the cage rim 1 = rat barely moving and no rising 0 = rat not moving at all
Forepaw outstretching	3 = symmetric forepaw outstretching 2 = right side moves, but less outstretching 1 = slight movement of the right forepaw 0 = no movement of the right forepaw
Climbing	3 = easy climbing and tight gripping of the wire 2 = right side is impaired or less tight gripping during climbing 1 = rat fails to climb or tends to circle
Body proprioception	3 = rat reacts by turning head and being startled by the stimulus on both sides 2 = slower reaction of the rat on the right side 1 = no response to the stimulus on the right side
Spontaneous walking activity	3 = rat walking straight ahead 2 = right circling 1 = rat tending to walk toward the right side 0 = rat not moving
Sensory function (vibrissae brushing)	3 = rat turns head towards the stimulus 2 = slow reaction to the stimulus on the right side 1 = no respond to the stimulus on the right side

^1^ minimum score = 3, maximum (best) score = 18.

**Table 2:** List of used primers in the study.

Primer		Sequence	bp ^1^	AT ^2^ [°C]
*Aim2*	s	tgctacggagctggtgtttt	186	62
	as	actccgtcctgtctgcaatg		
*CycloA*	s	ggcaaatgctggaccaaacac	196	65
	as	ttagagttgtccacagtcggagatg		
*Gapdh*	s	aacccatcaccatcttccag	196	60
	as	gtggttcacacccatcacaa		
*Nlrc4*	s	ggctgaggcccacgtataaa	98	60
s	as	ctcctctggctctctggact		

^1^ bp: basepairs; ^2^ AT: annealing temperature.

**Table 3:** List of used antibodies in the study.

Antibody	Company	Order Number	WB	IHC	IF
beta-Actin	Sigma Aldrich, Taufkirchen, Germany		1:5000	-	-
NLRC4	Merck–Millipore, Darmstadt, Germany	06-1125	1:5000	1:2000	1:200
AIM2	Bioss antibodies, Woburn, Massachusetts, USA	bs5986R	1:1000	1:300	-
NeuN	Merck–Millipore, Darmstadt, Germany	MAB377	-	-	1:500
IBA1	Merck–Millipore, Darmstadt, Germany	MABN92	-	-	1:600
GFAP	Abcam, Cambridge, UK	ab10062	-	-	1:1000
Donkey-anti-mouse 594	ThermoFisher Scientific, Waltham, Massachusetts, USA	A21203	-	-	1:500
Donkey-anti-rabbit 488	ThermoFisher Scientific, Waltham, Massachusetts, USA	A21206	-	-	1:500
Donkey-anti-mouse 488	ThermoFisher Scientific, Waltham, Massachusetts, USA	A21121	-	-	1:500
Goat-anti-mouse 546	ThermoFisher Scientific, Waltham, Massachusetts, USA	A21133	-	-	1:500

## Abbreviations

AIM2	absent in melanoma 2
AIS	acute ischemic stroke
ASC	Apoptosis-associated speck-like protein containing a CARD
DAMP(s)	damage-associated molecular patterns
E2	17β-Estradiol
GFAP	glial fibrillary acidic protein
Iba1	ionized calcium-binding adapter molecule 1
NACHT	NAIP (neuronal apoptosis inhibitor protein), C2TA (class 2 transcription activator, of the MHC), HET-E (heterokaryon incompatibility) and TP1 (telomerase-associated protein 1)
NeuN	Fox-3, Rbfox3, or Hexaribonucleotide Binding Protein-3
NLRC4	NLR family CARD domain-containing protein 4
NLRP3	NACHT, LRR and PYD domains-containing protein 3 or cryopyrin
OGD	Oxygen–glucose deprivation
P	Progesterone
PAMP(s)	pathogen-associated molecular patterns
tMCAO	transient Middle Cerebral Artery occlusion
TTC	2,3,5-triphenyltetrazolium chloride
